# Treatment of hypertrophic scars using fractional CO2 laser alone in comparison with adding botulinium toxin either intalesional injection or assisted drug delivery

**DOI:** 10.1007/s10103-026-05026-9

**Published:** 2026-10-02

**Authors:** Aya Fahim, Elham Saied, Marwa S. El-Mesidy

**Affiliations:** Kasr Al Ainy Faculty of Medicine, Department of Dermatology, Cairo University, Giza, Egypt

**Keywords:** Hypertrophic scar, BTXA, LADD

## Abstract

Burns result in hypertrophic scarring, causing functional impairment, cosmetic and psychological concerns. Fractional CO₂ laser and botulinum, have shown promise in treatment. To evaluate the effectiveness of fractional laser treatment alone and combined with botulinum toxin A, delivered either through injections or topical application as fractional laser-assisted drug delivery in hypertrophic burn scar. A randomized comparative trial involving 16 patients with post-burn hypertrophic scars. The scar of each patient was divided into 3 sections: (1) fractional CO₂ laser combined with intralesional botulinum toxin type A, (2) fractional CO₂ laser monotherapy, and (3) fractional CO₂ laser with laser-assisted drug delivery of BTXA. Patients received three treatment sessions at monthly intervals, followed by a three-month follow up. Clinical outcomes were evaluated using the Vancouver Scar Scale. Intralesional BTXA demonstrated superior efficacy, achieving the greatest reductions in VSS (− 58.25%). Intralesional BTXA in combination with fractional CO₂ laser represents a highly effective treatment for post-burn hypertrophic scars, offering superior improvements in scar pliability, contour, and patient comfort compared with laser monotherapy or LADD. These findings support intralesional BTXA as the preferred therapeutic approach, while highlighting the potential of combination protocols for optimizing scar outcomes.

## Introduction

Hypertrophic scars (HTS) are a significant clinical challenge resulting from a pathological, fibro proliferative abnormality in wound healing, causing considerable functional and psychosocial morbidity for patient [1]. Standard management involves diverse modalities, including intralesional corticosteroids and surgical revision, with laser therapy increasingly being used [2]. Fractional lasers create precisely controlled microchannels within the dermal scar tissue, inducing a targeted wound response driving remodeling and aiding in Laser-Assisted Drug Delivery (LADD) facilitating deep penetration of agents [3].

Botulinum Toxin Type A (BTX-A) helps in mitigation of wound tension, collagen modulation, and suppression of fibroblast proliferation and Connective Tissue Growth Factor (CTGF) expression [4]. Given BTX-A’s established efficacy and safety profile, its optimal delivery method in conjunction with fractional non-ablative laser treatment remains a critical area of investigation [5]. The current study evaluates the comparative efficacy of fractional ablative laser treatment combined with BTX-A delivered via intralesional injection versus topical application (LADD) for the resolution of hypertrophic burn scars.

## Patients and methods

Sixteen post-burn hypertrophic scars patients of both sexes were recruited from the dermatology outpatient clinic. Patients with bleeding disorders, allergies, neuromuscular junction diseases or receiving medications affecting neuromuscular transmission and pregnant and lactating women were excluded.

A randomized clinical study where each scar was divided into three distinct, equally-sized sections and marked with a marker before photographing. Given the intra-patient comparative design, each section within the same scar received a different treatment regimen. To ensure consistency and reduce potential bias, the allocation of treatment to specific scar sections was standardized in a fixed order as follows:

- **Sect.  1**: Received Fractional CO2 Laser with Intralesional Botox injection.
- **Sect.  2**: Received Fractional CO2 Laser Only.
- **Sect.  3**: Received Fractional CO2 Laser with Botulinum Toxin Assisted Drug Delivery (topical).

Scar evaluation was performed at baseline and 3 months after the last treatment session. All assessments were conducted for each of the three scar sections separately.

- **Photographic Evaluation and Documentation**

Standardized digital photographs were obtained for each scar section at baseline, before every session, and 3 months after the last session. Images were taken under consistent lighting, distance, and patient positioning to allow for objective comparison.

(b) **Vancouver Scar Scale (VSS)**

**VSS** assessed four key aspects of scar appearance: melanin pigmentation (0–2 points), scar height (0–3 points), vascularity (0–3 points), and pliability (0–5 points). Each component received a score, with a maximum total score of 13 points indicating the most severe scar formation.

### Treatment (Therapeutic intervention)

- Patients received 3 treatment sessions, scheduled every 4 weeks. Follow-up assessments were conducted 3 months after the last treatment session. Topical anesthesia (lidocaine 2.5%/prilocaine 2.5%) was applied to the working area under occlusion 30 min prior to each session.
- The lesion was sterilized with an alcohol swab before starting the procedure. An icepack was applied to the laser site for 10–20 min post-laser to soothe and reduce post-laser edema when needed, and before applying Botox.
- Fractional CO2 laser treatment was performed using (DEKA SmartXide DOT, Italy). Treatment parameters included power (18–20 W), dwell time (800–1000µs), spacing (500–600 μm, 13% density), micro spot size (120 μm), and 2–3 stacks, adjusted depending on scar height [6].

### Botulinum toxin

- Immediately following laser treatment, Botulinum toxin type A (Botox Allergan, Irvine, CA; 100U vacuum-dried powder reconstituted in 2mL of sterile, preservative-free 0.9% saline to a concentration of 5U/0.1mL) was injected intralesional into the scar tissue for Sect. 1.
- A 24-gauge needle (0.3 mm × 0.8 mm) was used, and the injection was continued until slight blanching was visible. The dose was adjusted to 2.5 U/cm³ of the lesion, not exceeding 100 units per session.
- Immediately following laser treatment for Sect. 3, topical Botulinum toxin (prepared at the same **concentration** as for intralesional injection and adjusted to 2.5 U/cm³) was applied to the treated area topically [7].

Patients were assessed every session for side effects, including pain, swelling, muscle weakness, infection, erythema, crustation, post inflammatory hyperpigmentation, hypopigmentation, pruritus, and scarring.

## Results

All patients completed the follow up period. Demographic data are presented in Table (1).

**Table 1:** Patient’s characteristics of the studied group

		Count	%
Gender	Male	12	75.0%
	Female	4	25.0%
Skin Type	III	7	43.8%
	IV	9	56.3%
Site of Burn (face or other)	Face	2	12.5%
	Another Site	14	87.5%
Site of Burn	Face	2	12.5%
	Retro-Auricular	1	6.3%
	Hand	1	6.3%
	Forearm	1	6.3%
	Arm	7	43.8%
	Back	3	18.8%
	Thigh	1	6.3%

The mean Vancouver Scar Scale (VSS) score decreased from 8.38 ± 0.89 to 3.50 ± 1.37 after treatment (*P* < 0.001), from 8.38 ± 0.89 to 4.44 ± 1.31 (*P* < 0.001) and from 8.38 ± 0.89 to 4.19 ± 1.47 (*P* < 0.001) in group 1, 2 and 3 respectively.

**Fig. 1:**
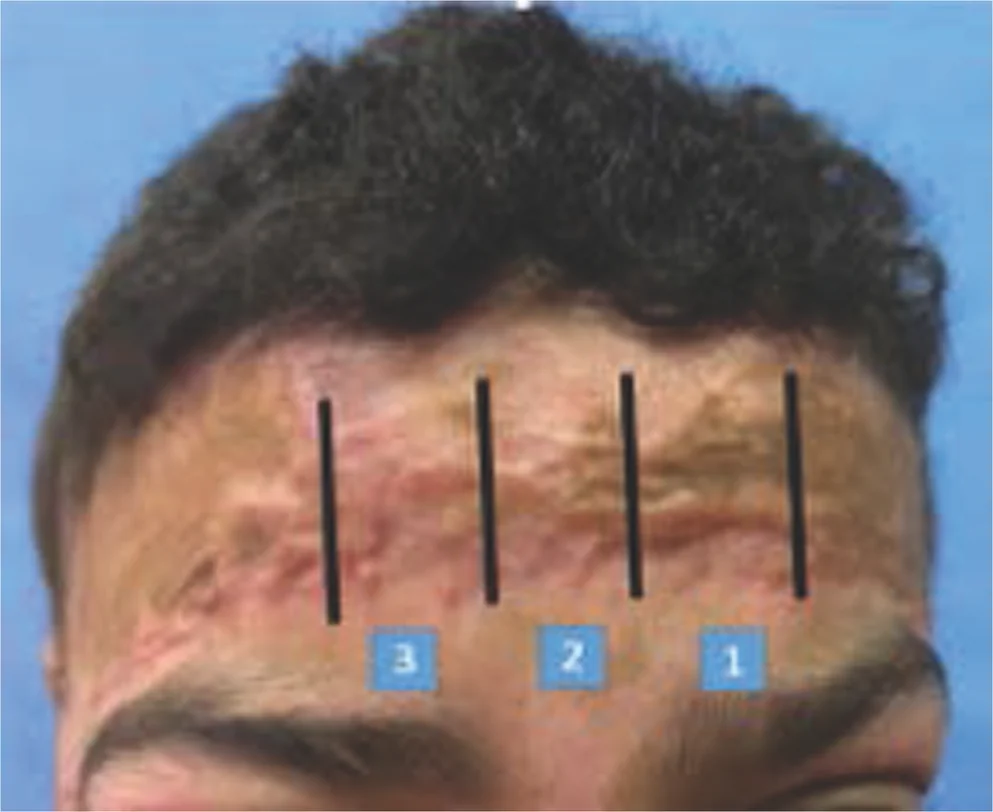
18-year old male patient with a hypertrophic burn scar showing VSS scores 9, 9 and 9 before treatment in section 1 and 2 and 3

**Fig. 2:**
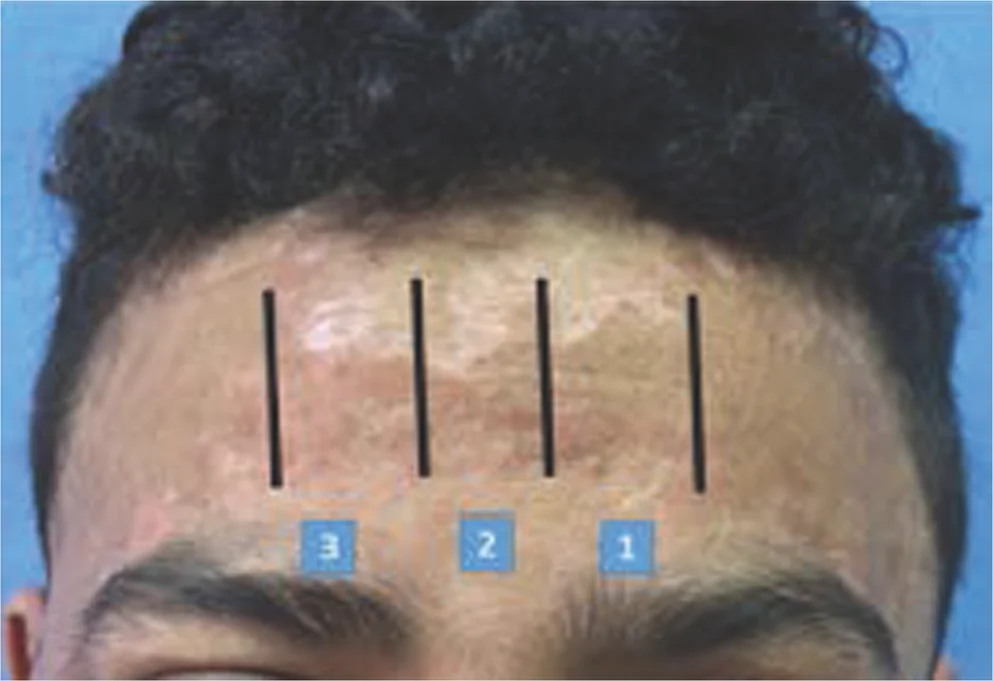
Note: the same patient showing VSS 1,2 and 2 after treatment in section 1 and 2 and 3 respectively

Group 1 demonstrated the greatest overall improvement in scar appearance, as reflected by the highest mean percentage change in VSS (− 58.25%). (Figs. 1 and 2).

Figures 1 and 2: 18-year-old male patient with a hypertrophic burn scar showing VSS scores of 9, 9 and 9 before treatment then 1, 2 and 2 after treatment in Sects.  1 and 2 and 3 respectively.

VSS assesses 4 items; pliability, pigmentation, vascularity and height. Regarding pliability percent change showed statistically significant differences between groups (*P* = 0.002), with Group 1 achieving the most substantial improvement (Mean = -71.3, SD = 18.5) compared to Group 2 (Mean = -48.2, SD = 22.3) and Group 3 (Mean = -54.9, SD = 23.1) as in Table (2).

**Table 2:** Comparison of VSS component changes between groups

		Group 1	Group 2	Group 3
VSS % change	Mean	-58.25	-47.08	-50.03
	SD	15.18	13.82	15.79
	*P* value	0.051	0.051	0.051
	*N*	16	16	16
Pigmentation % change	Mean	-22.0	-9.5	-9.5
	SD	39.2	20.1	20.1
	P value	0.742	0.742	0.742
	N	16	16	16
Vascularity % change	Mean	-52.1	-46.0	-43.8
	SD	43.5	49.8	50.4
	P value	0.856	0.856	0.856
	N	16	16	16
Pliability % change	Mean	-71.3	-48.2	-54.9
	SD	18.5	22.3	23.1
	P value	0.002	0.002	0.002
	N	16	16	16
Height% change	Mean	-44.0	-44.0	-31.0
	SD	51.0	51.0	47.9
	P value	0.385	0.385	0.385
	N	16	16	16

*P*-values represent between-group comparisons. Bold values indicate statistical significance (*p *< 0.05)

VSS total percent change showed the greatest improvement in Group 1 (Mean = -58.25, SD = 15.18) compared to Group 2 (Mean = -47.08, SD = 13.82) and Group 3 (Mean = -50.03, SD = 15.79), approaching statistical significance (*P* = 0.051) as in Table (2).

Blinded investigator scoring demonstrated differences across treatment groups when considering median values and interquartile ranges (IQR). Section  1 had a median of 3.0 with an IQR of 2.0. Section  2 showed a median of 2.5 with an IQR of 1.0. Section  3 a median of 2.0 with an IQR of 2.0 as in Table (3).

**Table 3:** Descriptive statistics

	Median	Mean	Std. Deviation	IQR	Range	Minimum	Maximum	25th percentile	75th percentile
Sect. 1	3.000	2.875	0.957	2.000	3.000	1.000	4.000	2.000	4.000
Sect. 2	2.500	2.438	0.964	1.000	3.000	1.000	4.000	2.000	3.000
Sect. 3	2.000	2.188	0.981	2.000	3.000	1.000	4.000	1.000	3.000

*QR* Interquartile Range

## Discussion

These inter-group findings highlight that while all three approaches produced measurable improvements, intralesional BTXA combined with fractional CO₂ laser provided the most consistent and statistically significant benefits in scar texture, pliability, and contour, establishing it as the most effective protocol compared with laser alone or laser-assisted topical delivery.

Efficacy of botulinum toxin A in treatment of scars could be explained as through several mechanisms; first it induces temporary paralysis on the wound muscle, leading to immobilization which plays an important role in decreasing the perpendicular tension on the wound edges, this perpendicular tension is responsible for impairing the process of normal wound healing. The second mechanism is through its action on collagen as reticular dermis prolonged inflammation can lead to increase in both collagen synthesis and glycosaminoglycans deposition, which in association of increased local metabolic activities can intensify hypertrophic scars. Botulinum toxin A has the ability to induce paralysis of the wound edges decreasing the tension applied on them until the collagen could mature. Finally, it can act through its effect on cell cycle and gene expression of fibroblasts as it can directly modulate fibroblast activity by altering apoptotic, migratory, and fibrotic pathways on pathological scars and thereby enhancing their appearance [8].

In addition to, direct injection ensures the drug reaches the fibroblast-rich dermis, where hypertrophic scar pathology originates. This deeper penetration achieves higher local concentrations and allows more consistent modulation of fibroblast activity and collagen remodeling. In contrast, topical BTXA, even when assisted by fractional CO₂ laser, penetrates mainly the superficial layers and shows variability in absorption, resulting in weaker effects on scar texture and contour [8].

Observations of the current study align with a study **on** 46 patients with benign skin tumors received postoperative injections of saline or BTXA at concentrations of 1, 2.5, or 5 U/0.1mL. Over six months, all BTXA groups achieved significantly higher modified Stony Brook Scar Evaluation Scale scores compared with saline controls [9].

Similarly, a large meta-analysis of 11 randomized clinical trials involving 486 cases, which concluded that Botulinum Toxin Type A (BTA) injection is a reliable and superior treatment for improving scar quality and wound healing in the face and neck area compared to placebo or control groups. Quantifiable results from this analysis demonstrated that the BTA group achieved significantly lower Vancouver Scar Scores, thinner scars, and higher patient satisfaction [10].

The results are also similar to randomized intra-patient comparative study, which investigated the use of intralesional Botulinum Toxin Type A (BTA) in 15 children with post-burn hypertrophic scars and keloids. The authors utilized split-lesion approach, injecting BTA into one part of the scar (with 5 IU/cm2) while leaving the other as a control. After six months of monthly treatments, the injected areas demonstrated dramatic and statistically significant VSS [11].

However, these results are different from a study conducted on 20 patients with Keloids and hypertrophic scars. One side was subjected to intralesional injection of botulinum toxin type A once a month for 4 months with a dose of 2.5 U/cm^3^ of the lesion, not exceeding 100 units per session and the other side was subjected to four sessions of CO_2_ laser therapy at 1 month interval followed by topical application of botulinum toxin A. Significant improvement was noted in Vancouver Scar Scale in hypertrophic scars in laser group (LADD) than intralesional botulinum toxin A. This difference may be explained by the fact that previous studies used intralesional BTX injections alone without combining them with fractional CO₂ laser [7].

Fractional CO₂ laser monotherapy itself plays an important role in scar treatment by inhibiting fibroblast proliferation, stimulating the release of basic fibroblast growth factor (b-FGF), which reduces collagen synthesis, and decreasing TGF-β1 activity, which otherwise promotes collagen production. These biological effects are triggered by deep tissue ablation, which initiates a cascade of heat shock proteins, delays collagen synthesis and regeneration, and accelerates wound healing [12].

Klosová et al. (2021) reported that Fractional CO_2_ laser therapy alone has achieved statistically significant improvement of the texture and reduction of hypertrophic scars and overall improvement of functional and esthetic result. Another meta-analysis in 2021, demonstrated that fractional CO_2_ laser therapy was valuable for reducing burn scar severity [13].

Combination therapy significantly enhances scar improvement by integrating complementary mechanisms of action. Intralesional Botulinum Toxin A (BTA) is uniquely effective in optimizing scar texture and pliability, while the LADD method facilitates improved clinical appearance by enhancing the vascularity, leading to a better overall total hemoglobin level and distribution and subsequent reduction in erythema. This idea aligns with a retrospective study including 128 patients with hypertrophic scars (56 underwent monotherapy defined as fractional CO2 laser and topical growth factors, and 72 underwent combined therapy defined as sequential combination of BTX-A, fractional CO2 laser, and topical growth factors, ), which were followed-up for up to 15 months after the initiation of treatment to collect demographic and clinical data. The analysis showed that the combined therapy significantly outperformed monotherapy in improving Vancouver scar scale scores (*P* < 0.05) and in the reduction of scar thickness (*P* < 0.05), without increasing adverse complications. This proves that combination approaches offered superior efficacy [5].

Analysis of the Vancouver Scar Scale (VSS) demonstrated statistically significant differences in pliability between treatment groups, with the intralesional BTXA arm achieving the greatest softening of scar tissue, thereby confirming its superiority in contour remodeling.

These findings align with prior report Klosová et al. (2021) emphasizing that BTXA’s primary benefit in tissue pliability and collagen remodeling [14].

Blinded investigator scoring demonstrated notable differences across treatment groups when considering median values and interquartile ranges (IQR). Area 1 (fractional CO_2_ laser with intralesional BTXA) showed the highest median score of 3.0 with an IQR of 2.0, reflecting consistently stronger outcomes across patients. Area 2 (fractional CO_2_ laser alone) had a median of 2.5 and a narrower IQR of 1.0, indicating moderate improvement with less variability. Area 3 (fractional CO_2_ laser with laser-assisted topical BTXA) recorded the lowest median of 2.0 and an IQR of 2.0, suggesting greater variability and comparatively weaker performance. These findings highlight the superior and more consistent results achieved with the intralesional BTXA–laser combination compared to the other modalities.

## Data Availability

No datasets were generated or analysed during the current study.

## References

[1] -Wasson E, Thandi C, Hitchens E, Thomas K et al (2023) An audit of the use of fractional CO2 laser for hypertrophic burn scars: a real-life perspective. Lasers Med Sci 38(1):144. 10.1007/s10103-023-03814-1 37347311

[2] Sheng M, Chen Y, Li H, Zhang Y et al (2023) The application of corticosteroids for pathological scar prevention and treatment: current review and update. Burns trauma 11:tkad009. 10.1093/burnst/tkad009 PMC10025010 36950503

[3] -Shilova M, Plummer K, Ware R, Kimble R et al (2026) Laser-Assisted Drug Delivery for Hypertrophic Scar Treatment: A Scoping Review. J burn care research: official publication Am Burn Association 47(1):130–146. 10.1093/jbcr/iraf167 PMC12770983 40971504

[4] Raslan EE, Bakhamees BH, Howsawi TA, Alshmrani LS et al (2024) The Efficacy of Botulinum Toxin Type A (BTA) in the Treatment of Hypertrophic Scars and Keloids: A Systematic Review and Meta-Analysis of Randomized Controlled Trials. Cureus 16(10):e71161. 10.7759/cureus.71161 PMC11548679 39525161

[5] -Wang J, Huang L, Li J, Xu R et al (2024) Efficacy and safety of sequential treatment with botulinum toxin type A, fractional CO2 laser, and topical growth factor for hypertrophic scar management: a retrospective analysis. Sci Rep 14(1):27233. 10.1038/s41598-024-78094-y PMC11549097 39516539

[6] -Tawfic SO, El-Tawdy AM, Shalaby S, El-Khalawany MA et al (2020) Evaluation of fractional CO_2versus long-pulsed Nd:YAG lasers in hypertrophic scars and keloids: A randomized clinical trial. Lasers Surg Med 52(10):959–965 10.1002/lsm.23249 32293045

[7] -Sabry HH, Abdel-Motaleb AA, Shalaby SM (2020) Assessment of laser-assisted delivery versus intralesional injection of botulinum toxin A in treatment of hypertrophic scars and keloids. Dermatol Ther 33(6):e13980 10.1111/dth.13980 32638463

[8] -Kasyanju Carrero LM, Ma WW, Liu HF, Yin XF et al (2019) Botulinum toxin type A for the treatment and prevention of hypertrophic scars and keloids: Updated review. J Cosmet Dermatol 18(1):10–15 10.1111/jocd.12828 30548742

[9] -Shi H, Zhang P, Zhang J, Sun J et al (2024) Dose-dependent effects of botulinum toxin type A on prevention of postoperative scars in various regions in the body: A prospective, double-blind randomized controlled trial. Aesthetic Plast Surg 49(3):862–874 10.1007/s00266-024-04351-0 39218835

[10] -Guo X, Song G, Zhang D, Jin X (2020) Efficacy of botulinum toxin type A in improving scar quality and wound healing: A systematic review and meta-analysis of randomized controlled trials. Aesthetic Surg J 40(5):NP273–NP285 10.1093/asj/sjz165 31155638

[11] -Tawfik AA, Ali RA (2023) Evaluation of botulinum toxin type A for treating post burn hypertrophic scars and keloid in children: An intra-patient randomized controlled study. J Cosmet Dermatol 22(4):1256–1260 10.1111/jocd.15634 36718819

[12] -Waibel JS, Wulkan AJ, Shumaker PR (2013) Treatment of hypertrophic scars using laser and laser assisted corticosteroid delivery. Lasers Surg Med 45(3):135–140 10.1002/lsm.22120 23460557

[13] -Klosová H, Xinopulos P, Zálešák B, Lamgová (2021) K.Fractional CO2 laser therapy of hypertrophic scars - evaluation of efficacy and treatment protocol optimization. Acta Chir Plast 63(4):171–180 10.48095/ccachp2021171 35042360

[14] Khatery BHM, Hussein HA, Abd-El-Raheem TA, Hanbuli E, H. M., Yassen NN (2022) Assessment of intralesional injection of botulinum toxin type A in hypertrophic scars and keloids: Clinical and pathological study. Dermatol Ther 35(10):e15748 10.1111/dth.15748 36190006

